# Influence of Guidance on Occupational Image and Traineeship’s Satisfaction of Vocational Students

**DOI:** 10.1007/s12186-023-09341-y

**Published:** 2024-01-03

**Authors:** Annie Dubeau, Yves Chochard

**Affiliations:** Département d’éducation et de formation spécialisées, Université du Québec à Montréal, C.P. 8888, succursale Centre- Ville, Montréal, Québec H3C 3P8 Canada

**Keywords:** Traineeship, Occupational Image, Guidance, Traineeship Supervisor, Vocational Training

## Abstract

Initial vocational training (VT) in high school consists of short-term programs leading to employment in a skilled trade. To better align training with employment opportunities and to encourage students to stay in the programs until they graduate, most programs include traineeship. Since traineeships involve acquiring skills directly on the job, they require greater involvement of supervisors to guide the trainees. Given the importance of on-the-job guidance in achieving traineeship objectives, this study examines the potential influence of three dimensions of guidance provided by traineeship supervisors –planning, support, and training– on students’ job perception (i.e., occupational image) and traineeship satisfaction. Overall, the results provide mixed results, partially supporting the mediation hypothesis suggested by the results of previous studies. Indeed, the results reveal that the quality of the training offered by the supervisor affects subsequent students’ satisfaction with traineeship experience. Training has an indirect effect on satisfaction via the occupational image held by students. However, the expected indirect links between the other two dimensions of supervisor guidance –degree of planning and support perceived by the student– and the students’ image of their chosen occupation could not be confirmed. The results support the importance of providing quality on-the-job training to students during their studies.

## Introduction

According to United Nations Educational, Scientific and Cultural Organization (UNESCO) terminology (UNESCO Institute for Statistics, 2013), initial high school vocational training (VT) is at the “upper secondary education” level. In the United States and Canada, these programs are mostly taught in trade schools or through traineeship programs and typically provide well-structured training. In Québec, Canada, where this study was conducted, it includes short programs of study (ranging from 600 to 1,800 h) that can be completed in two years or less and that provide the specific hands-on skills required to work in a trade such as medical secretary, travel agent, lineman, elevator mechanic, baker, welder, and firefighter. Application requirements to these programs are not overly restrictive. For example, in Quebec, Canada, to be admitted, applicants must be at least 16 years old on September 30 of the school year the program begins and have obtained Secondary III or IV (Grade 9 or 10) credits in language of instruction, second language, and mathematics (*Loi sur l’instruction publique* RLRQ c. I-13.3, r. 10 n.d.). Therefore, Quebec’s VT system is accessible to a wide range of prospective students. However, many VT students suffer from psychosocial difficulties (e.g., anxiety, depressive symptoms and problematic substance use) and struggles balancing work, school and family which, combined with the intensive structure of their training, pose significant threats to their education success and graduation (Beaucher et al., 2021). To ensure that most students persevere until the end of their program and are well-prepared to handle the job they have been trained in, their learning environment must meet their needs in class and during traineeships.

Most VT programs in Québec include several traineeships to better align training with employment opportunities and to encourage students to stay in the programs until they graduate. Traineeships are on-the-job work experiences that serve several purposes. They give vocational students the role of trainee that has some similarities with the role of apprentice. Trainees has the opportunity to observe experienced workers, apply skills developed during classroom training, and acquire new skills directly on the job. All of these endpoints allow student to validate and develop their perceptions about practicing the trade and easier integrate into the workplace (Giret & Issehnane, 2012; Hardy & Parent, 2003). To give this guidance and strong involvement of traineeship, supervisors are required (Mazalon et al., 2014). The guidance during traineeship is likely to influence trainees’ perception of their future occupation and their overall satisfaction experiences with the subsequent traineeships included in their curriculum (Giret & Issehnane, 2012).

The purpose of this article is to study the potential influence of three dimensions of guidance provided by traineeship supervisors –planning, support, and training– on the trainees’ occupational image and traineeship satisfaction. This, in order to open avenues of reflection and action on the elements that impact people’s decisions to follow and persevere in VT and traineeships.

Because of the complexity of the trade’s tasks and working conditions, studies have shown that traineeships can be a challenging experience for trainees (e.g., Doray & Bastien 2015; Gagnon, 2020; Mazalon et al., 2014; Veillette 2004). Indeed, studies have concluded that the main causes of trainees dropping out of a VT program are poor working conditions during traineeship (Lamamra & Masdonati, 2009; Losa et al., 2014). For example, a qualitative study of future Swiss bricklayers found that traineeships conditions are sometimes considered difficult by trainees (Duemmler et al., 2022). In this study, various difficulties such as a high-level stress caused by the production rate, the privation of time for learning new tasks, and a lack of tolerance for mistakes by superiors who expect trainees to work as fast as experienced workers are reported. Lack of guidance from supervisors can exacerbates these poor working conditions during the traineeship and cause a negative occupational image of the chosen vocation. In turn, a negative occupational image of the chosen vocation may affect the traineeship satisfaction for subsequent traineeships included in their VT program. (Veillard, 2012; Veillette, 2004). As Veillard (2012) points out, the complexity of workplace situations implies that complete self-learning is insufficient. Supervisors’ guidance is essential to ensure that the traineeship serves as opportunities for trainees to learn and shape their professional identity; and that the latter, is not solely based on one specific work context (Darrah, 1996; Eraut, 2007; Veillard, 2012).

Despite the role of guidance during the traineeship, little is currently known about the correlates and outcomes associated with traineeship guidance among the population of VT students. This is especially important in light of the significant skilled labour shortage currently happening in Canada (Statistics Canada, 2022; Institut du Québec [IDQ] 2021) and many other developed countries around the world (Eurofound, 2021).

## Theoretical Background

According to Akkerman and Bakker (2012), traineeships should be an integral part of vocational programs. In this regard, Billett (2001, 2004) has shown that workplace affordances and appropriate guidance increased the trainees’ engagement and learning during traineeships. In short, for the traineeship to be successful, workplace supervisors must provide appropriate guidance that will give trainees a positive image of the vocation they are pursuing.

### Traineeship Guidance

In-service guidance means supporting and coaching trainees in work situations while they learn about the different techniques of the trade (Caprani et al., 2022). To conceptualize the guidance provided in traineeships, we drew on three dimensions: traineeship planning, student support, and student training (Beaulieu et al., 2021).

First, traineeship supervisors perform traineeship planning tasks by explaining to students the objectives and the tasks they are expected to perform in their work placement and how the placement will unfold. They also ensure that trainees have the material resources required to successfully complete their traineeship (Billett, 2004). According to Kunégel (2011), during the traineeship planning, supervisors engage in three key activities: task selection, providing instructions, and what he calls "pre-guidance". The supervisors play a pivotal role in carefully selecting the task to assign to the trainees. In the task selection, the supervisors consider various factors, including the task's complexity, the presumed skill level of the trainees, production requirements, and the supervisors’ commitment to the trainees’ training. When the supervisors explain the task at hand, it's not merely about providing instructions. This is referring to contextualizing the instructions, defining the boundaries of the action, specifying the level of autonomy the trainees have executing the task, offering valuable insights for successful execution, instilling confidence, and actively involving the trainees in the assigned on-the-job task. At this juncture, supervisors must carefully consider the trainees’ unique skills and abilities. Furthermore, the supervisors’ approach involves ensuring that the trainees find their own solutions without the burden of excessive guilt in case of setbacks. This entails selecting tasks that strikes a delicate balance: it should be challenging for the trainees (i.e., enough to foster learning and growth but not so daunting as to overwhelm the trainee) and do not cause too much loss of productivity to the company –in the case that the trainees could not correctly carry out the requested task–. While conveying instructions, supervisors also assume a "pre-guidance". This function is designed to thoroughly equip the trainee for the upcoming task, with the primary aim of minimizing errors and mitigating risk. It entails offering the trainees a series of precise recommendations pertaining to the activity in question –such as demonstrating the correct procedure for tightening screws to avoid wheel locking– and explaining the key indicators of a successful execution (for example, ensuring the wheel exhibits no signs of resistance).

The second dimension relates to student support. The focus on this dimension lies on fostering independent work among the trainees. In this case, supervisors play a mentoring role; to demonstrate certain tasks to the trainee, and then observe, monitor, and correct them. Traineeship supervisors also tailor the level of complexity of the tasks to the level of the trainees (Mazalon et al., 2014). They provide advice and share their expertise with the trainees (Billett, 2004). As noted by Kunégel (2011), the level of autonomy granted to the trainees is adjusted based on their learning requirements. While trainees engage in their tasks, the effective supervisors maintain a form of “remote supervision” and occasionally providing a support to facilitate and enhance the trainees' skills development.

Finally, supervisors oversee student training. This dimension relates to the idea that workplace supervisors must define the context in which the traineeship will be conducted, that is, identify the actual work situations in which the students’ skill(s) will need to be developed (Tessier et al., 2017). The role of the traineeship supervisors is therefore to identify situations in the productive activities of the host workplace that can provide rich learning opportunities, and then set the duration and sequencing of these activities in the students’ program (Roy et al., 2017). For Kunégel (2011), the training is closely linked to the trainees’ actions. During training, supervisors are intended to guide the gesture, orient the action, prevent errors, and provide useful information for execution. All the on-the-job tasks are performed by the trainees under the supervisor watchful eye. Kunégel (2011) distinguishes two modes of training: instrumental training and advisory-based training. Instrumental training comes into play when trainees are assigned to tasks that exceed their current skill levels. In such instances, workplace supervisors take charge of the critical tasks while trainees observe. Supervisors lead and demonstrate the task's execution, while trainees observe or engage in secondary tasks (e.g., setting up diagnostic equipment or printing error messages). In advisory-based training, trainees perform the tasks under the watchful eyes of the workplace supervisors who offer guidance if needed. This guidance encompasses various aspects, including the quality of work, client safety, and self-preservation. Throughout the training process, supervisors evaluate the trainees’ progress. As per Kunéguel (2011), this evaluation takes on various forms, such as assessing the trainees’ knowledge and competence levels (e.g., through questioning to gauge is understanding), providing constructive feedback (both positive encouragement and constructive criticism), and conducting post-task debriefings with the trainees to reflect on recent events, including any errors that may have occurred.

Such workplace supervisors’ guidance will help the trainees build a positive image of the vocation they are pursuing and allows them to enjoy their subsequent traineeships (Brillet & Gavoille, 2016).

### Occupational Image of the Chosen Vocation and Traineeship Satisfaction

Vocational choices, and hence the choice for or against the vocational program, seem to rely a lot on mental images. The concept of occupational image, like other types of images (brand image, self-image), is known to influence people’s behaviour (Brillet & Gavoille, 2016). According to those authors, “Occupational image is a holistic representation of an occupation in the mind of an individual. It refers to the set of mental representations formed as a result of an individual’s exposure to different internal and external stimuli” (p. 58, translated by author). It concerns the satisfaction of carrying out the work or tasks related to the occupation, belonging to this group of workers, and joining the companies that hire such workers (Osty, 2008). The influence of occupational image on the behaviour of individuals can be considered at different times, such as when choosing a program of study or when completing a traineeship. A positive occupational image is likely to promote attraction and retention for those in the profession (Guichard & Huteau, 2006; Huteau, 1982). It is therefore plausible that a favourable occupational image has a positive influence on subsequent students’ traineeships satisfaction included in the VT program (Brillet & Gavoille, 2016). A negative image would have the opposite effect. Indeed, during a traineeship, the trainees confront their image of the trade with the reality of the trade (Lamamra & Masdonati, 2009). An appropriate guidance during the traineeship allows the trainees to positively improve their occupational image and to be more satisfied with their subsequent experiences of traineeship.

**Fig. 1:**
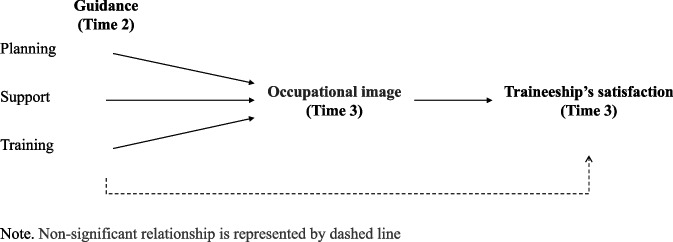
Path Evaluated

In sum, previous studies have established the influence of supervisors’ guidance on occupational image. As Veillard (2012) observes, workplace learning is not without its constraints. Notably, it may lead to the acquisition of suboptimal work techniques. Hence, it is crucial to provide appropriate guidance during internships and to offer multiple internship opportunities in various settings or teams. This approach aims to enhance the learning process and the trainees' perception of the profession they are pursuing. Furthermore, the occupational image has an impact on the satisfaction derived from the traineeship experience (Brillet & Gavoille, 2016). As illustrated in Fig. 1, traineeship guidance indeed has an impact on subsequent occupational image and satisfaction with the traineeship experiences included later into the curriculum.

Broadly speaking, the caliber of guidance provided by traineeship supervisors is reported to have an impact on trainees, particularly in shaping their occupational image. For its part, occupational image has been demonstrated to significantly enhance the trainees’ overall satisfaction with their traineeship experiences (Brillet & Gavoille, 2016).

## This Study

To test these propositions (see Fig. 1), a longitudinal study including three measurement times was conducted with students in a VT program. More precisely, we postulated that the supervisors’ guidance provided (i.e., traineeship planning, student support, and training) as measured after their first traineeship would positively predict the students’ occupational image at the end of their program (Hypothesis 1). We also postulated that students’ occupational image would mediate the relationship between supervisors’ guidance in previous traineeship and satisfaction with traineeship (Hypothesis 2). While testing these hypotheses, we controlled for the effects of school motivation, as measured at the beginning of the VT program (Time 1), on traineeship satisfaction given that previous studies have repeatedly shown that school motivation affects school satisfaction and achievement (Authors; Eccles & Wigfield, 2020; Morris et al., 2021). This study will provide important insights regarding the consequences of supervisors’ guidance during traineeship in VT programs.

## Methodology

### Participants

The sample consisted of 192 students (43% female; 57% male) aged 16 to 62 (*M*: 23.92; *SD*: 9.07), registered in a one-year VT program. All students were enrolled in one of the 13 professional training centers that agreed to participate in the study. The training centers were mainly attended by students from various rural and suburban towns in the province of Quebec, Canada.

### Procedure and Measures

Students who participated in the study completed a questionnaire assessing the selected variables three times in class. The principal investigator met with all groups of students 1) at the beginning of their program (Time 1, Fall 2017); 2) after their first traineeship (Time 2, Winter 2018); 3) at the end of their VT program (Time 3, Spring 2018). After receiving information about the study and completing the consent form to participate in the study, the students individually completed the questionnaire, which lasted a maximum of 20 min. If required, the principal investigator answered students’ questions to ensure smooth progress of data collection. Participants indicated their responses to items in the questionnaire using a 7-point Likert scale, and a 10-point Likert scale for traineeship satisfaction.

*Traineeship guidance*. The perception of guidance provided to the students by the traineeship supervisor was evaluated using the French-language scale *Échelle de mesure de la qualité des stages* (QSMT) [Traineeship Quality Scale] composed of 16 items. The scale included three subscales validated with French-speaking VT students in Quebec (Beaulieu et al., 2021). The traineeship planning subscale (6 items) measured the quality of traineeship organization and preparation (α = 0.93). The subscale included items such as “The supervisor discusses the objectives of my traineeship with me.” The student support subscale (5 items) assessed students’ perceptions of practices that foster skills development in the workplace (α = 0.85). The subscale included items such as “During the traineeship, I have the opportunity to use a variety of skills.” Finally, the third subscale consisted of 6 items (α  = 0.85) assessing student training. The scale included items such as “The traineeship supervisor gives me regular feedback on my learning and progress” that measured the extent to which students had access to tasks and activities appropriate to their skill level.

*Occupational image.* A nine-item scale was used (α = 0.73) to assess students’ perception of the social status of their chosen occupation and their satisfaction with their choice of occupation (Watt  & Richardson, 2007). An example item from this scale is “How happy are you with your decision to enter this occupation?”.

*Traineeship satisfaction* was measured using an item phrased, “How satisfied were you with your traineeship?” Participants were asked to indicate their choice on a scale of 1 to 10, with 1 being the lowest level of satisfaction and 10 being the highest.

*Motivation.* As mentioned earlier, we controlled for student achievement motivation at the beginning of the training program (Time 1). Student achievement motivation was derived from two scales evaluating students’ self-efficacy beliefs and task values. The two scales were originally developed by Eccles and Wigfield (1995). The French versions of these scales were validated with French-speaking students by Dubeau et al. (2017). The measure of self-efficacy included 5 items (α = 0.61) assessing the extent to which the students felt capable of successfully completing the various tasks and courses of the study program. The scale included items such as “I think I will be able to deal with the demands of my study program.” The task values scale comprised 14 items (α = 0.81), each reflecting one of four dimensions: importance, interest, cost, and perceived utility value of the training program. An example item was “I attach importance to doing well this year.”

### Analyses

Path analyses were performed using the MPlus software (Muthén et al., 2017). The maximum likelihood estimation method was used, and all analyses were performed on the variance–covariance matrix to test the fit of the data to the proposed theoretical model. As suggested by experts in this type of analysis, the validity of the proposed model was examined using several indices. As such, the chi-square (χ ^2^) test, the number of degrees of freedom (*df*), the Bentler-Tucker-Lewis coefficient (TLI; Hu & Bentler, 1999), the comparative fit index (CFI; Bentler, 1990), the root mean square error of approximation (RMSEA; Ullman, 2007), and the standardized root mean square residual (SRMR; Hu & Bentler, 1999) were used. The chi square is a test of the level of discrepancy between the fitted covariance, as specified in the hypothesized model and the sample covariance. A finding of non-significance corresponds to an adequate model. In addition, various authors suggest that a χ ^2^/dl ratio of less than 3 (Kline, 2016), and TLI and CFI values greater than 0.90 (Hu & Bentler, 1999; Ullman, 2007), reveal a good model fit. Furthermore, RMSEA values of less than 0.05, and SRMR of less than 0.08, signify a good model fit to the data (Ullman, 2007). Our mediational hypothesis (i.e., the indirect links between quality of guidance and overall traineeship satisfaction) was tested by performing bootstrap analyses that simulated 2,000 samples (Preacher & Hayes, 2008). The bootstrapping method tests whether the indirect relationship between a predictor variable and the outcome variable is significant in the context of a model that controls for multiple covariates (Cheung & Lau, 2008).

## Results

Descriptive statistics and correlations are presented in Table 1.

**Table 1:** Descriptive Statistics and Correlations for All Variables Assessed in the Study

	Mean (SD)	1	2	3	4	5
Guidance						
1. Planning	5.58 (1.52)	…				
2. Support	5.88 (1.08)	.83***	…			
3. Training	5.22 (1.52)	.78***	.70***	…		
4. Occupational image	4.82 (0.95)	.46***	.38***	.37***	…	
5. Traineeship’s satisfaction	8.26 (2.21)	.60***	.44***	.56***	.55***	…

^***^ *p* < .001

**Fig. 2:**
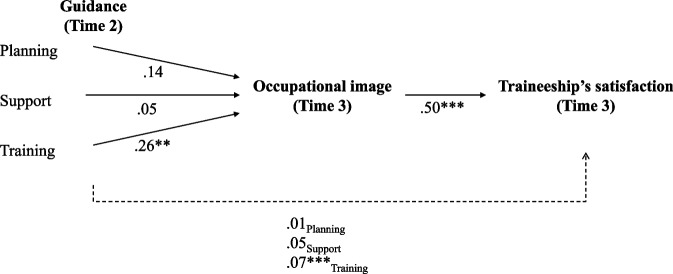
Final Path with Maximum Likelihood Estimates (standardized estimates). Non-significant Relationships are Represented by Dashed Lines. **p < .01, ***p < .001. Co-variance Terms Were Included between the Three Dimensions of Guidance. All Error Terms were Significant at *p* < .001

The results of the initial model tested with trainees concluded that it adequately represented the data, since the fit indices met the standards set, χ ^2^(4) = 4.68, *p* < 0.322; TLI = 0.99; CFI = 1.0; RMSEA 0.03 [0 – 0.12]; SRMR = 0.03. This model was thus considered as final. Results are presented in Fig. 2.

As shown in Fig. 2, the quality of guidance provided to the students during their traineeship at Time 2 predicted the degree of satisfaction with another traineeship included in their program (i.e., at Time 3). This relationship was mediated by the students’ image of the occupation they were training for (at Time 3). However, our mediation hypothesis is confirmed only for one of the three dimensions of guidance included in the theoretical model, namely, the training received from the traineeship supervisor (Table 2).

**Table 2:** Bootstrapping Results to Test for the Indirect Relationships Between Planning, Support and Training at T2, and Traineeship’s Satisfaction at T3

	Traineeship’s satisfaction
Planning	β = .07; *p* < .01; CI: -.01; .18
Support	β = .07; *p* < .01; CI: -.017; .192
Training	β = .14; *p* < .01; CI: .03; .28

Bootstrap analyses included 2000 samples; confidence intervals (CI) were set to 95%

Indeed, for the training dimension, zero is excluded from the confidence interval for the indirect effect, so it is significant. There is mediation. It is not the case for the other two guidance dimensions (planning and support).

## Discussion

The purpose of this study was to examine the potential influence of three dimensions of guidance provided by traineeship supervisors – traineeship planning, student support, and the training provided – on trainees’ perceptions of their chosen occupation (occupational image) and their satisfaction with the traineeship, which were subsequently measured at the end of their VT program. The mediation hypothesis that was postulated suggested that the workplace supervisor’s guidance provided to trainees during their traineeship would indirectly affect, through their occupational image, their subsequent appreciation of other traineeships provided during their program. Overall, the results provided mixed results, partially supporting the mediation hypothesis suggested by the literature review.

It was possible to empirically confirm the mediation hypothesis to explain the relationship between the training provided by the supervisors during the traineeship and the trainees’ appreciation of other traineeships included in their program. Indeed, the results of the study revealed that when the training provided by the supervisors in the first traineeship affected the students’ subsequent satisfaction with the traineeships, it had an indirect effect on this variable via the occupational image held by the students at the end of their VT. Specifically, traineeship satisfaction was indirectly influenced by the quality of training provided to the students by the traineeship supervisors at the beginning of the program. However, the expected indirect links between degree of traineeship planning and support perceived by the students and their occupational image could not be confirmed by the results of this study. Overall, these results are consistent with previous studies that postulate that the quality of guidance provided by supervisors contributes to the students’ positive images of their chosen occupation and their appreciation of other traineeships included in their program (Veillard, 2012). These findings suggest that interventions that specifically target the quality of training provided by traineeship supervisors appear to be a relevant avenue for improving students’ satisfaction with their traineeship experiences. Indeed, working upstream before the start of the traineeship to help and train workplace supervisors to provide effective training is likely to promote the development of a positive occupational image and enhance students’ satisfaction with their traineeship experiences later in the training process. On the other hand, planning the onboarding of students and providing them with timely support while they carry out their traineeship activities appear to be good training practices (Gagnon, 2020). Although not all dimensions of guidance seem to have delayed effects over time, as planning and support do not appear to contribute to students developing a positive image of the occupation they are training for and to influence their satisfaction with their traineeships later in their VT program, this study highlights that supervisor guidance is significant beyond its immediate context of action, i.e., during the traineeship. Traineeship guidance extends beyond mere task execution within the specific workplace setting where the traineeship occurred because supervisors' interventions have a substantial impact, contributing to the development of trainees' professional identities and influencing their future traineeship and work-related pursuits. This is especially pertinent in societies, like the United States and Canada, that prioritize immediacy and the present moment. While the primary objective of on-the-job training is to enable trainees or new employees to carry out their tasks effectively, workplace supervisors, through their guidance, wield influence not only over job acquisition but also in shaping the professional growth and long-term satisfaction of their employees and trainees, including their occupational perceptions.

Based on these findings, it is important to pay close attention to workplace training since it has effects that go beyond the traineeship experience itself. It is particularly important because labour shortages mean that companies are now hiring young people without degrees and training them “on the job” (Chochard et al., 2023). Improving on-the-job training will result in better quality activities for both new workers and students doing traineeships as part of their academic programs. Similar studies including these variables would also be needed to assess the generalizability of these findings.

### Limitations and Research Avenues

This study has some limitations that should be considered in future research. First, the results are based on a convenience sample, which limits the possibility of generalizing these results to the entire VT population. It would therefore be desirable to replicate the results of this study using other samples and in other countries. Moreover, the use of a larger sample would make it possible to consider the potential influence of more variables, including school engagement, academic performance, and obtaining employment instead of doing traineeship; this would contribute to improving our understanding of the role of guidance during traineeships and beyond. Another limitation is the use of self-reported instruments to measure the study variables. This contributes to increasing the risk of social desirability. To overcome this limitation, more studies using other types of objective measurements obtained either from mentors or by direct observation from the researcher would be needed. This would provide an alternative perspective on variables that reflect students’ perceptions. In spite of these limitations, this study is innovative in proposing a longitudinal analysis. The use of data collected at different times during the academic program made it possible to evaluate the effects of training on other students’ perceptions. In this sense, the study confirms the importance of providing trainees and new workers with quality on-the-job training.
